# Bulgarian population – before COVID-19 and now: Dima Tsanova

**DOI:** 10.1093/eurpub/ckac131.063

**Published:** 2022-10-25

**Authors:** D Tsanova, Ts Valentinova, Ts Vitkova, K Statev, El Mineva-Dimitrova, A Seizov

**Affiliations:** Bulgarian Public Health Association, Faculty of Public Health, MU-Pleven, Pleven, Bulgaria

## Abstract

**Background:**

The demographic picture in Bulgaria became worse and worse since the democratic changes at the end of the 20th century. Our country was at one of the first positions according to the level of death rate and the rate of population decline. The study aims to analyse the effect of the COVID-19 pandemic on the main demographic indicators in Bulgaria.

**Methods:**

Data from Bulgarian National Statistical Institute were used to analyse population growth and life expectancy and to calculate the death rate, birth rate, infant mortality rate, and the share of people over 65 years of age for 2019 in comparison with 2021.

**Results:**

In 2019 before the onset of COVID-19, the Bulgarian population consists of 6 951 481 people. In 2021 it declines to 6 838 937 people. The decrease in birth rate is not very significant - from 8.9‰ /2019/ to 8.5‰ /2021/. The analysis provides estimates of excess deaths observed during the peak of the COVID-19 outbreak in Bulgaria. The death rate is very much increased - from 15.5‰ to 21.7‰. The increase affects the female and male populations equally - from 19.6‰ for men in 2019 to 23.2‰ in 2021 and from 16.4‰ for women to 20.2‰. The life expectancy shows a certain decrease - from 74.8 years /2019/ to 74.64 /2021/. Correspondingly, life expectancy was reduced for females- by 78.34 - 78.22 and for males by 71.37 - 71.11 years. The level of infant mortality is not changed - 5.6‰. People over 65 years are 21.6% of the total population during the compared period.

**Conclusions:**

The Bulgarian population is very strongly affected by COVID - 19 pandemic. The COVID-19 pandemic has caused a significant number of deaths worldwide but Bulgaria ranks first in the world in terms of mortality rates. Life expectancy decline reflects the impact of temporary epidemic mortality. The impact on children from the pandemic is not very significant for the country. Society should be making major and cost-effective efforts to reduce mortality.

**Key messages:**

• The sharp change in demographic realities has significant effects on the country’s economy.

• The deterioration of natural growth exacerbates the need to increase labour productivity in areas with the fastest declining populations.

